# Predictive and concurrent validity of pain sensitivity phenotype, neuropeptidomics and neuroepigenetics in the MI-RAT osteoarthritic surgical model in rats

**DOI:** 10.3389/fcell.2024.1400650

**Published:** 2024-08-08

**Authors:** Colombe Otis, Katrine-Ann Cristofanilli, Marilyn Frezier, Aliénor Delsart, Johanne Martel-Pelletier, Jean-Pierre Pelletier, Francis Beaudry, Bertrand Lussier, Alexandre Boyer, Eric Troncy

**Affiliations:** ^1^ Research Group in Animal Pharmacology of Quebec (GREPAQ), Université de Montréal, Saint-Hyacinthe, QC, Canada; ^2^ Osteoarthritis Research Unit, University of Montreal Hospital Research Center (CRCHUM), Saint-Hyacinthe, QC, Canada; ^3^ Département de Biomédecine Vétérinaire, Faculty of Veterinary Medicine, Université de Montréal, Saint-Hyacinthe, QC, Canada; ^4^ Centre Interdisciplinaire de Recherche sur le Cerveau et L’apprentissage (CIRCA), Université de Montréal, Montreal, QC, Canada

**Keywords:** miRNA, epigenetic, musculoskeletal, chronic nociplastic pain, quantitative sensory testing

## Abstract

**Background:**

Micro-RNAs could provide great insights about the neuropathological mechanisms associated with osteoarthritis (OA) pain processing. Using the validated *M*ontreal *I*nduction of *R*at *A*rthritis *T*esting (MI-RAT) model, this study aimed to characterize neuroepigenetic markers susceptible to correlate with innovative pain functional phenotype and targeted neuropeptide alterations.

**Methods:**

Functional biomechanical, somatosensory sensitization (peripheral–via tactile paw withdrawal threshold; central–via response to mechanical temporal summation), and diffuse noxious inhibitory control (via conditioned pain modulation) alterations were assessed sequentially in OA (*n* = 12) and Naïve (*n* = 12) rats. Joint structural, targeted spinal neuropeptides and differential expression of spinal cord micro-RNAs analyses were conducted at the sacrifice (day (D) 56).

**Results:**

The MI-RAT model caused important structural damages (reaching 35.77% of cartilage surface) compared to the Naïve group (*P* < 0.001). This was concomitantly associated with nociceptive sensitization: ipsilateral weight shift to the contralateral hind limb (asymmetry index) from −55.61% ± 8.50% (D7) to −26.29% ± 8.50% (D35) (*P* < 0.0001); mechanical pain hypersensitivity was present as soon as D7 and persisting until D56 (*P* < 0.008); central sensitization was evident at D21 (*P* = 0.038); pain endogenous inhibitory control was distinguished with higher conditioned pain modulation rate (*P* < 0.05) at D7, D21, and D35 as a reflect of filtrated pain perception. Somatosensory profile alterations of OA rats were translated in a persistent elevation of pro-nociceptive neuropeptides substance P and bradykinin, along with an increased expression of spinal miR-181b (*P* = 0.029) at D56.

**Conclusion:**

The MI-RAT OA model is associated, not only with structural lesions and static weight-bearing alterations, but also with a somatosensory profile that encompasses pain centralized sensitization, associated to active endogenous inhibitory/facilitatory controls, and corresponding neuropeptidomic and neuroepigenetic alterations. This preliminary neuroepigenetic research confirms the crucial role of pain endogenous inhibitory control in the development of OA chronic pain (not only hypersensitivity) and validates the MI-RAT model for its study.

## 1 Introduction

Osteoarthritis (OA) affects more than 25% of the population in Western countries, ranking it as the most common degenerative joint disease (GBD, 2018), with its prevalence rising yearly due to global aging and obesity. This complex disease, involving joint structural damage and evolving pain, challenges therapeutic development. Biochemical changes contribute to nociceptive peripheral sensitization (Malfait and Schnitzer, 2013). Increased nociceptive inputs may lead to centralized sensitization, an extended hyperexcitability of central nervous system (CNS) pain circuits, and an adaptive CNS response (Malfait and Schnitzer, 2013; Eitner et al., 2017). Individuals with advanced OA may experience pain from nociceptive, inflammatory mechanisms, excessive (neuropathic) excitability, and/or deficient endogenous inhibitory control in pain processes (Fu et al., 2018). Classically characterized by local cartilage degeneration, bone remodeling, synovium inflammation, and soft-tissue alterations (Dobson et al., 2018), OA establishes a complex pain process described as nociplastic (Fitzcharles et al., 2021; Buldys et al., 2023), involving chronic pain with increased sensitivity due to altered function in pain-related sensory pathways (Chimenti et al., 2018).

The translation from experimental pain animal models to effective clinical treatment for chronic pain faces significant challenges, with some attributing these translation failures to certain shortcomings in clinical trials, or the lack of validity in animal models and/or chronic pain assessment methods, which might hinder the translation of promising interventions (Mogil, 2017). The chemical intra-articular injection of monosodium iodoacetate (MIA) is the most common OA pain model in rats, and in this model, central sensitization may be associated with an up-regulation of spinal neuropeptides, indicating the activation of peripheral nociceptors on peptidergic afferent C-fibers (Im et al., 2010; Eitner et al., 2017). However, criticism has emerged regarding the etiopathogenesis, acute occurrence, and temporal transience of this OA pain model, which may not necessarily be related to the OA disease, classifying it more as inflammatory and nociceptive (Barve et al., 2007; Hummel and Whiteside, 2017; Otis et al., 2017).

Various surgical models of OA have been tested in rats (Gervais et al., 2019). The one that combines cranial cruciate ligament transection (CCLT) and destabilization of the medial meniscus (DMM) associated with an exercise protocol, known as the *M*ontreal *I*nduction of *R*at *A*rthritis *T*esting (MI-RAT) model, has been demonstrated to allow the progressive development of structural OA over time and indicates persistent chronic pain changes (Otis et al., 2023). These changes include behavioral biomechanical alterations, sensory mechanical hypersensitivity, and spinal neuropeptide changes (Gervais et al., 2019; Keita-Alassane et al., 2022; Otis et al., 2023). In a recent study leading to the refinement of the surgical CCLT – DMM OA model, it was observed that gender dimorphism must be carefully considered when evaluating OA pain, as 17β-estradiol supplementation influenced central sensitization development (Keita-Alassane et al., 2022). Interestingly, associating calibrated slight exercise (on a treadmill) with stifle instability surgical induction in the MI-RAT model resulted in major benefits, including 1) homogenization of structural alterations and 2) persistence of the pain and sensory sensitization profile over time, resembling the human OA condition more closely (Otis et al., 2023). The sensitization level was lower than in sedentary CCLT – DMM group and implied the involvement of endogenous inhibitory control (EIC), as supported by spinal neuropeptidomics.

In continuation of the previous studies, this work aims to advance the development of quantitative sensory testing (QST) of pain in association with neuropeptidomics and neuroepigenetics. Validated in human patients, QST is a psychophysical test method investigating the functional state of the somatosensory system (Mucke et al., 2021). It assesses sensory (pain) loss (hypoalgesia, reinforcement of EIC) and sensory (pain) gain (hyperalgesia/allodynia). Static QST was previously validated concurrently with neuropeptidomics, to be related to somatosensory hypersensitivity, using the MIA rat chemical OA model (Otis et al., 2016; Otis et al., 2017; Gervais et al., 2019; Otis et al., 2019), the surgical CCLT and/or DMM OA models (Gervais et al., 2019; Keita-Alassane et al., 2022), as well as in the MI-RAT OA model (Otis et al., 2023). Dynamic QST, allowing exploration of the altered function of pain-related sensory pathways in the periphery and CNS, i.e., facilitation or inhibition of pain signals, were adapted, in the current study, for testing in the MI-RAT model.

Epigenetic mechanisms regulate gene expression without altering the primary DNA sequence. Endogenous small non-coding single-stranded RNA, defined as micro-RNAs (miRNAs), plays pivotal role in post-transcriptional gene regulation of a wide range of biological processes (McDonald and Ajit, 2015). They modulate gene expression by binding to target mRNAs, affecting translation (Lu, 2023), or degradation, and are involved in diverse cellular processes, including development, differentiation, disease pathways, and are suspecting to play an important role in chronic pain (Lutz et al., 2014; Golmakani et al., 2024). Considering that alterations in protein expression play a crucial role in the development of long-term hyperexcitability in nociceptive neurons and contribute to chronic pain establishment, miRNAs hold great potential for elucidating nociceptive sensitization processes (Gold and Gebhart, 2010). Understanding miRNA involvement could unravel novel targets for managing chronic pain and mitigating nociceptive sensitization. To our knowledge, only one study has demonstrated altered expression of spinal miR-146a and the miR-183 cluster, linked to OA pain in the stifle joint, in a surgically induced OA rat model (Li et al., 2013).

The hypothesis of this preliminary study was that neuroepigenetics might be modulated by somatosensory sensitization development (or vice-versa) in an animal model of chronic OA pain. Taken together, the expression of neuroepigenetics, neuropeptidomics, and pain phenotype, particularly QST, would highlight pathophysiological mechanisms at the peripheral, spinal, and/or supraspinal levels, recognized to be involved in nociplastic pain (Buldys et al., 2023). In a prospective, randomized, blinded, and controlled study, the objectives were to document parallel changes induced in the MI-RAT OA model on spinal neuroepigenetics and neuropeptidomics in the non-evoked expression of musculoskeletal pain, as well as on evoked QST. This would pursue the determination of the MI-RAT as a predictive and concurrently validated translatable OA model.

## 2 Results

### 2.1 Functional pain outcomes

#### 2.1.1 Static weight-bearing (SWB)

**FIGURE 1:**
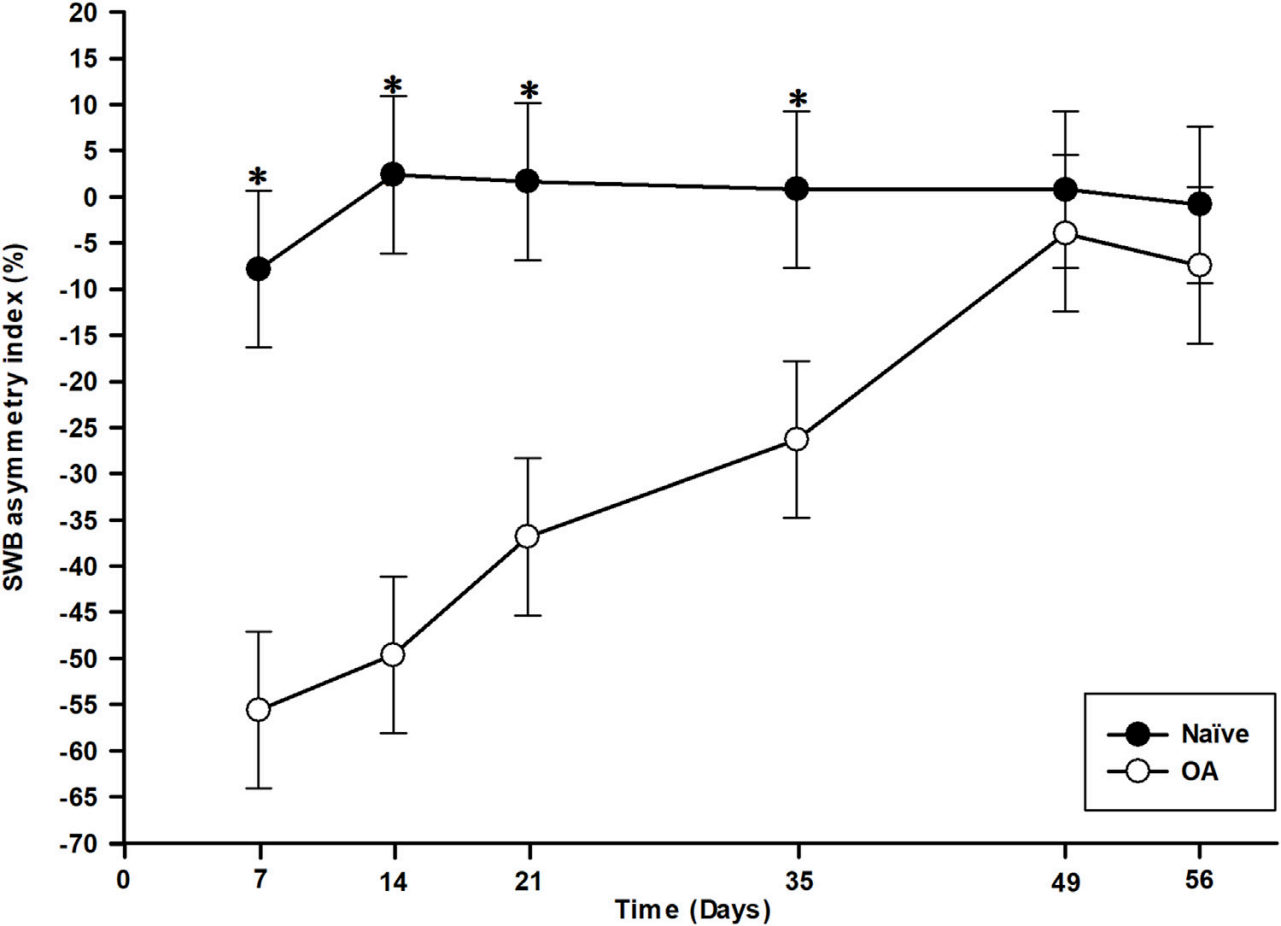
Temporal evolution of static weight-bearing (SWB) asymmetry index in the osteoarthritis (OA) and the Naïve rat groups. Temporal evolution of SWB asymmetry index (%) from the ipsilateral (right) to the contralateral (left) hind limb (least square means ± 95% confidence intervals). The OA group presented a marked contralateral report of SWB from D7 to D35 compared to the Naïve group (*P* < 0.001). The contralateral transfer then disappeared at D49 and D56 (*P* > 0.280). *Inter-group significant difference at each day (*P* < 0.050).

The contralateral weight shift from the right (OA-induced) to the left hind limb is represented as the SWB asymmetry index in Figure 1. Statistical analysis (general linear mixed model) revealed significant effects of group (*P* < 0.001), time (*P* < 0.001), and time *×* group interaction (*P* < 0.001). Rats in the OA group exerted a markedly higher SWB report to the left hind limb following OA induction. From day (D)7 to D35 inclusively, the SWB report in the OA group was statistically significant and different in comparison to the Naïve group (*P* < 0.001), indicating that OA rats applied less weight on their affected limb (right hind limb) from the first timepoint of assessment and reported it on their contralateral limb (left hind limb). The SWB asymmetry index (least square means ± 95% confidence intervals) in the OA group ranged from −55.61% ± 8.50% at D7 to −26.29% ± 8.50% at D35, demonstrating a statistically significant temporal change (*P* < 0.001). Over time, results in Figure 1 suggest that OA rats gradually reduced the weight applied from the contralateral to the ipsilateral hind limb, leading to a normal SWB distribution at D49 (*P* = 0.438) and D56 (*P* = 0.283) with no significant difference compared to the Naïve group.

#### 2.1.2 Static QST: tactile paw withdrawal threshold (PWT)

**FIGURE 2:**
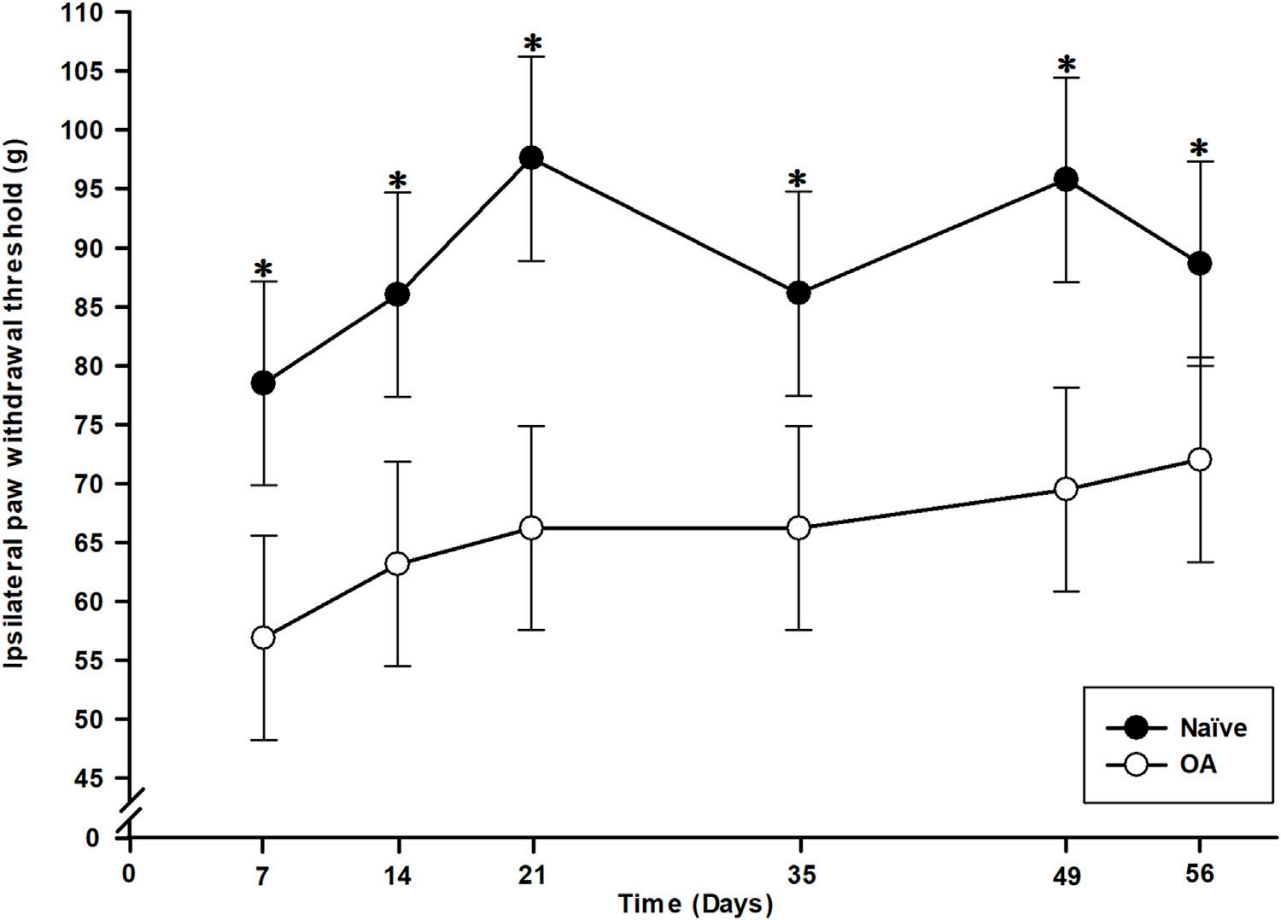
Temporal evolution of the paw withdrawal threshold (PWT) of ipsilateral (right) hind paw in the osteoarthritis (OA) and the Naïve rat groups. Temporal evolution of the ipsilateral PWT in grams (least square means ± 95% confidence intervals). A significant mechanical pain sensitivity in ipsilateral paw was present as soon as D7 in OA group and maintained up to D56 (*P* < 0.008) supported by lower PWT. *Inter-group significant difference at each day (*P* < 0.050).

Statistical analysis (general linear mixed model) of right hind PWT values revealed significant effects of group (*P* < 0.001), time (*P* = 0.009), although there was no time *×* group interaction effect (*P* = 0.617). Hence, the OA rats presented lower PWT (*i.e.*, evoked mechanical pain hypersensitivity) in the ipsilateral (right) paw (Figure 2) throughout the entire study period (D7 to D56) compared to the Naïve group (*P* < 0.008). The PWT for the OA group remained with a significant (*P* < 0.021) lower threshold than baseline up to the end of the experiment.

#### 2.1.3 Dynamic QST: response to mechanical temporal summation (RMTS) facilitation and conditioned pain modulation (CPM) inhibition

**FIGURE 3:**
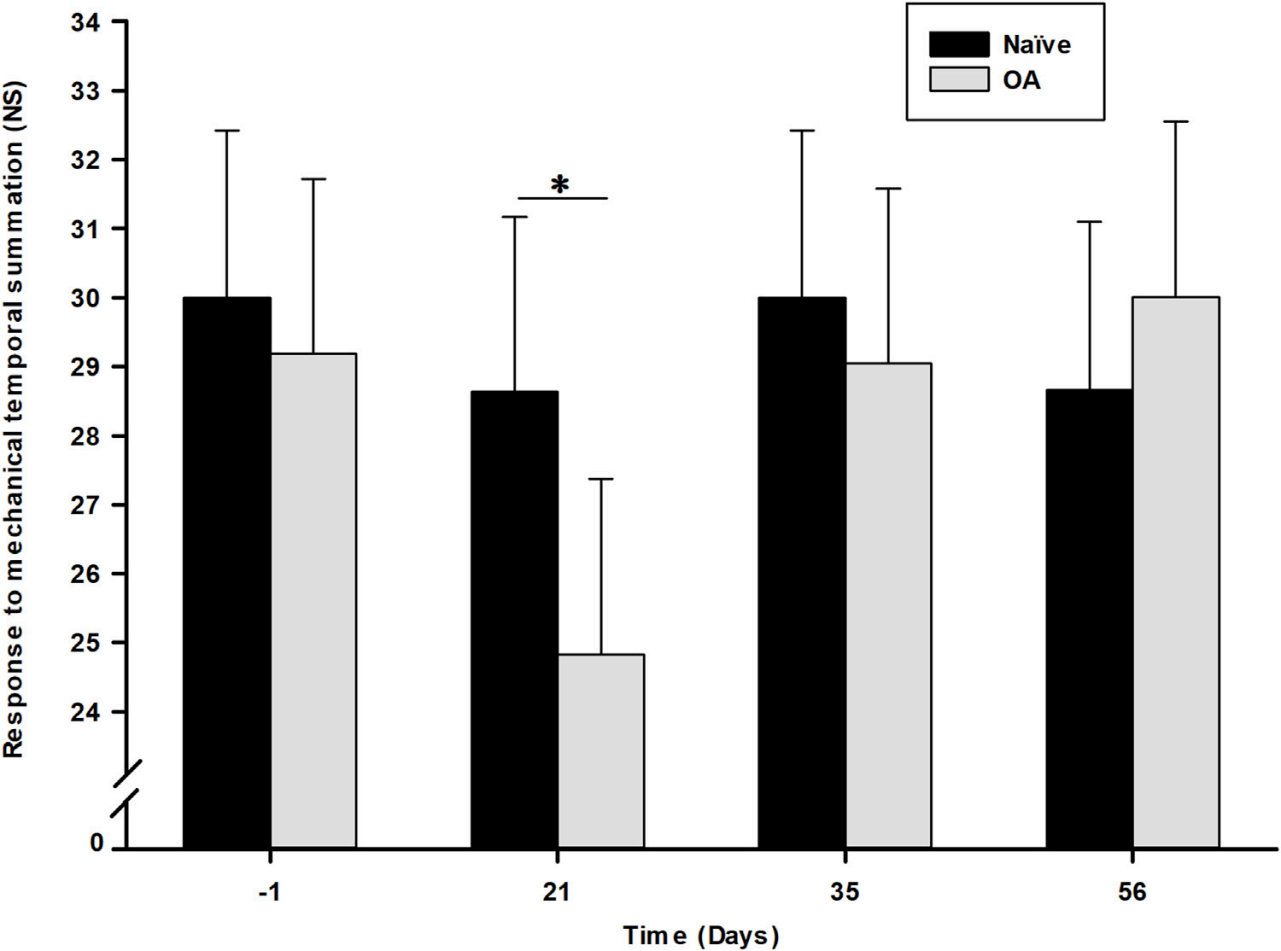
Dynamic quantitative sensory testing evolution overtime of response to mechanical temporal summation as number of stimuli (NS) required to induce a response in the Naïve and osteoarthritis (OA) groups. Dynamic QST is expressed in number of stimuli (NS) needed to induce a pain behavioral response (least square means ± 95% confidence intervals), with a cut-off of 30 NS. Central sensitization was noted at D21 (*P* = 0.038) in the OA group by a significant decrease in NS to induce a withdrawal reactive behavior. *Inter-group significant difference at each day (*P* < 0.050).

The RMTS number of stimuli required to induce a behavioral pain/discomfort-expressive response related to central sensitization development after repeated mechanical stimulation is illustrated in Figure 3. Statistical analysis (general linear mixed model) demonstrated only a significant effect of time (*P* = 0.045), with no significant impact observed for the group (*P* = 0.298) or the time *×* group interaction (*P* = 0.231). However, a significant decrease in the number of stimuli necessary to trigger a behavioral response was noted in the OA group (24.27 ± 2.53) at D21 (*P* = 0.038) compared to the Naïve group (28.64 ± 2.53). No significant inter-group difference was observed at D35 (*P* = 0.590) and D56 (*P* = 0.448) between both experimental groups.

**FIGURE 4:**
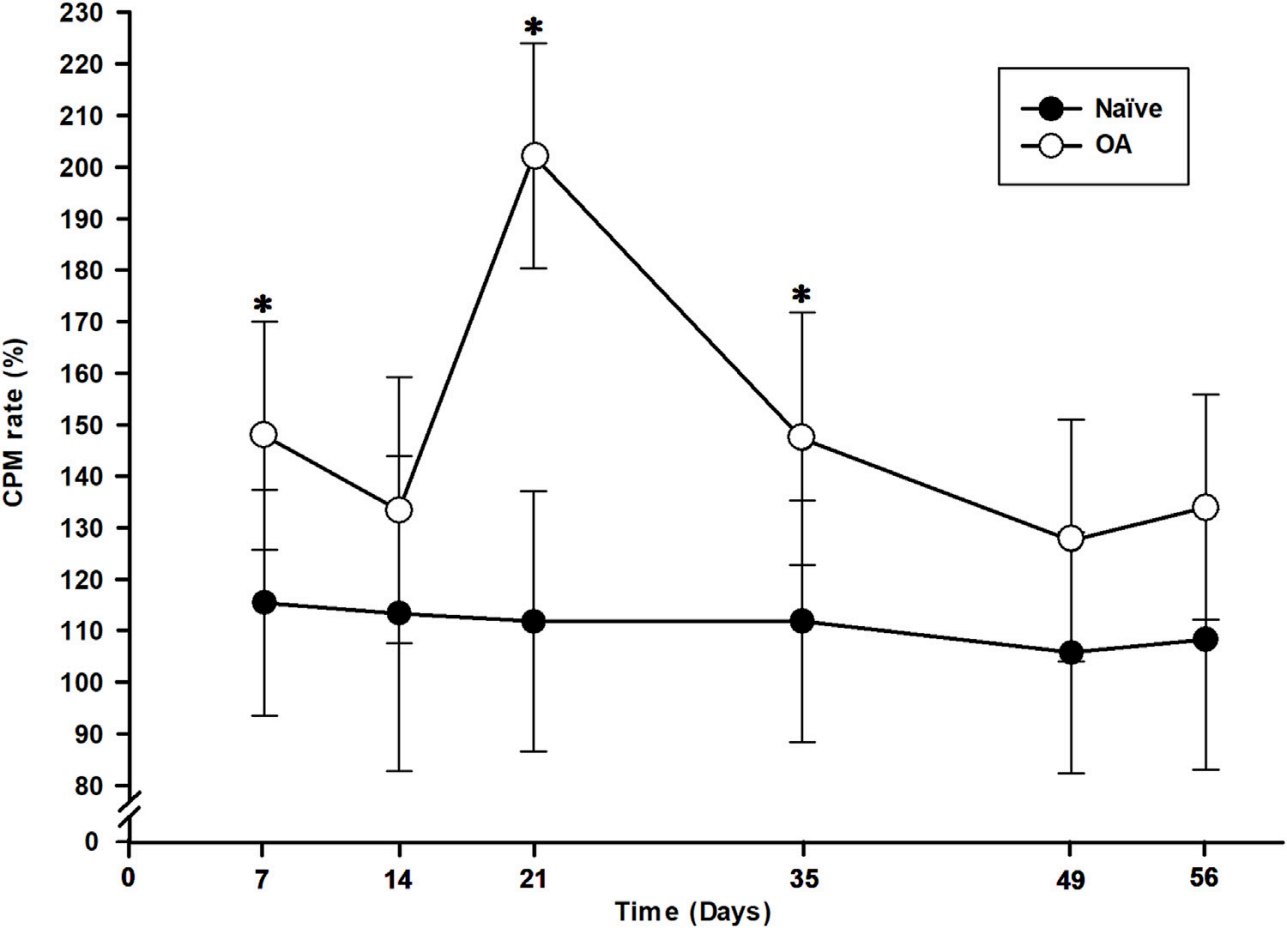
Temporal evolution of conditioned pain modulation (CPM) in the osteoarthritis (OA) and the Naïve rat groups. The temporal evolution of CPM rate (in percentage of increase in delta CPM post-*minus* pre-CS PWT) for each group (least square means ± 95% confidence intervals) was determined by including positive responders of CPM in the ipsilateral right hind paw. An increase in CPM rate is a measure of nociceptive endogenous inhibitory control activation. CPM rate of OA rats was higher at D7, D21 and D35 (*P* < 0.050) and significantly different than Naïve rats (*P* < 0.001). *Inter-group significant difference at each day (*P* < 0.050).

The measure of EIC activation following a dynamic conditioning stimulus (CS) is represented by the CPM functional rate in Figure 4 for both the OA and Naïve rat groups. The percentage of CPM positive responders in each group at all timepoints is presented in Table 1. Compared to baseline, there was a trend from D14 up to D56 of the number of CPM positive responders to increase in the OA group, with a statistically significant inter-group difference (Fisher’s exact test) at D21 (*P* = 0.037). In addition, the ipsilateral right hind CPM PWT functional rate (general linear mixed model) exhibited significant group (*P* < 0.001) and time effects (*P* = 0.001), without a time *×* group interaction (*P* = 0.088). A substantial increase in the CPM functional rate for OA rats (Figure 4) was present throughout the entire follow-up period (group effect). Specifically, significant increases were observed at D7 (32.43%, *P* = 0.050), D21 (90.37%, *P* < 0.001), and D35 (35.37%, *P* = 0.050) compared to the CPM functional rate of Naïve rats.

**TABLE 1:** Percentage of positive responders to conditioned pain modulation (CPM)**.**

Experimental group	Days						
	−1	7	14	21	35	49	56
Naïve (%)	67.68	100.00	67.68	58.33	83.00	58.33	83.00
OA (%)	75.00	67.68	100.00	100.00	72.73	90.91	90.91
*P*-value of group comparison	1.000	0.093	0.093	**0.037**	0.317	0.155	1.000

Bold value highlights statistical significance.

### 2.2 Molecular analysis

#### 2.2.1 Comparison of spinal neuropeptides revealed an increase in the concentration of pain-related neuropeptides in the OA group compared to the Naïve group

**FIGURE 5:**
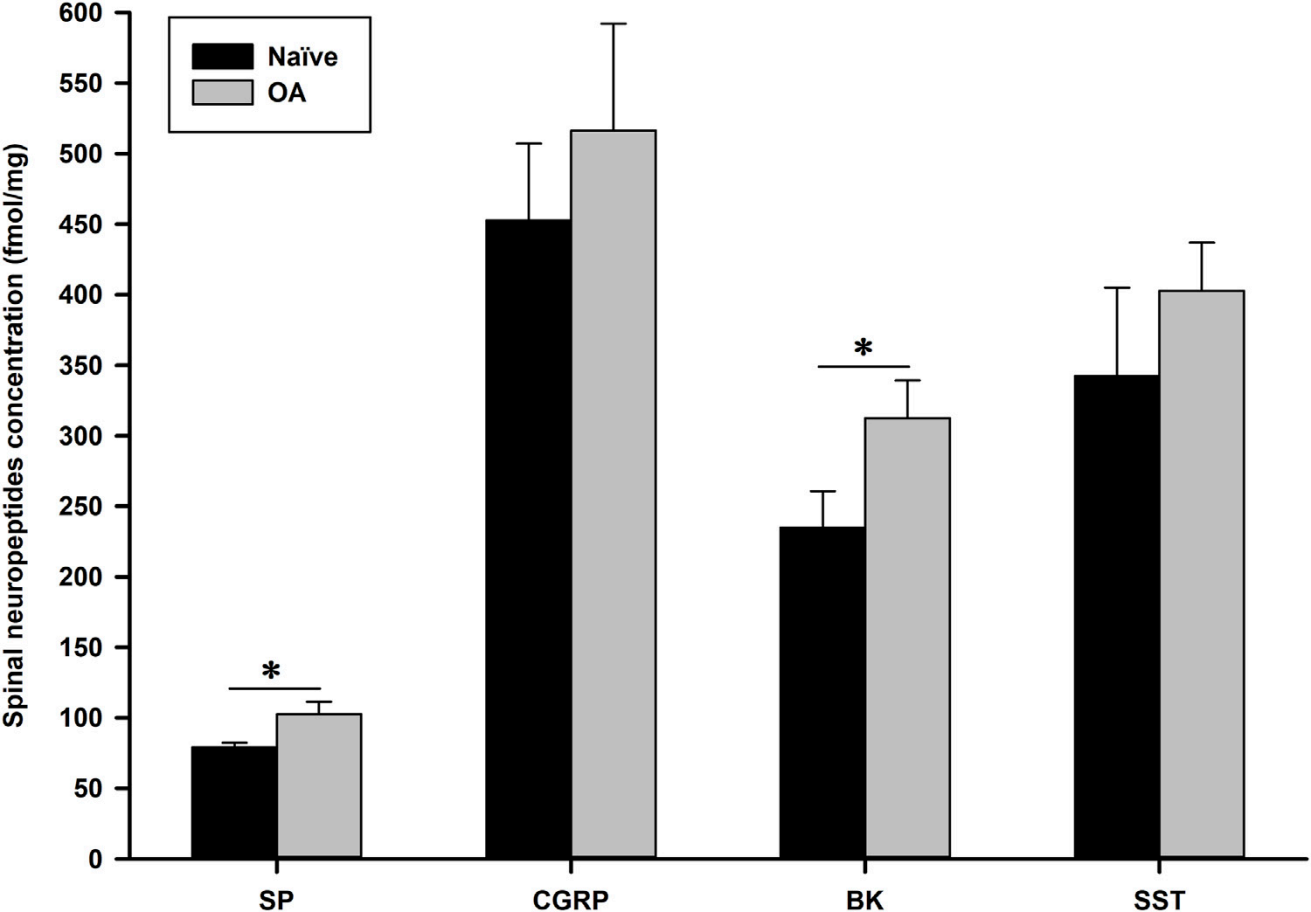
Spinal neuropeptides concentration (fmol/mg) of substance P (SP), calcitonin gene-related peptide (CGRP), bradykinin (BK) and somatostatin (SST) 56 days after induction of the MI-RAT model. The MI-RAT OA model induced a significant increase at D56 in SP and BK spinal cord (mean ± standard deviation) of OA rats (*P* = 0.002). CGRP and SST were also higher in OA rats, however, not at a statistically significant level (*P* = 0.093 and 0.065, respectively). *Inter-group significant difference for each neuropeptide (*P* < 0.050).

At D56, in comparison (two-sided Mann-Whitney-Wilcoxon test) to Naïve-ovariectomized rats, the OA group exhibited a significant (*P* = 0.002) increase in spinal concentration (mean (standard deviation); median [min-max]) of substance P (SP) (102.59 (8.72); 102.40 [91.90–115.25] fmol/mg) *versus* (79.16 (3.20); 79.90 [74.96–82.99] fmol/mg) and bradykinin (BK) (312.61 (26.28); 312.74 [272.95–354.31] fmol/mg versus 235.04 (25.60); 237.88 [187.27–262.30] fmol/mg), as illustrated in Figure 5 (data values are represented as mean (standard deviation)). The other two neuropeptides, calcitonin gene-related peptide (CGRP) and somatostatin (SST), displayed a similar trend of increase in OA rats (516.33 (75.84); 497.99 [447.37–664.80] and 402.73 (34.28); 419.90 [345.86–429.36] fmol/mg, respectively) compared to Naïve-ovariectomized rats (452.82 (54.31); 451.69 [370.31–532.39] and 342.47 (62.47); 324.55 [295.46–466.14] fmol/mg, respectively), although the difference was not statistically significant (*P* = 0.093 and *P* = 0.065, respectively).

#### 2.2.2 Comparison between the OA and Naïve groups for the expression level of fourteen selected pain-related miRNAs showed a difference in the expression of one miRNA

Analysis (two-sided Mann-Whitney-Wilcoxon test) of the data revealed that 56 days after the OA induction (surgical joint instability) and development (through regular calibrated slight exercise), miRNA expression in the OA rat spinal cord differed for one of the fourteen analyzed miRNAs (Table 2). Indeed, only miR-181b-5p showed a statistically significant alteration in its expression (*P* = 0.029) compared to the Naïve group.

**TABLE 2:** Fold change (OA/Naïve) in expression of selected miRNAs for RT-qPCR screening in spinal cord 56 days after OA induction and development.

miRNA	Condition (*Tissue*)	References	^a^Fold change OA/Naïve	^b^ *P*-value
miR-181b-5p	Inflammatory pain OA knee and facet joint Myalgic encephalomyelitis Chronic visceral pain (SDH; 2,100 miRNAs screened in blood)	Sengupta et al. (2013), Song et al. (2013), Nepotchatykh et al. (2020)	**1.26**	**0.029**
miR-1-3p	Neuropathic pain Inflammatory pain Cancer pain (DRG; SDH)	Kusuda et al. (2011), Bali et al. (2013)	1.14	0.818
miR-21-5p	Neuropathic pain Inflammatory pain (DRG; CNS; exudates)	Recchiuti et al. (2011), Bhalala et al. (2012), Sakai and Suzuki, 2013	1.13	0.699
miR-219a-2-3p	Inflammatory pain (*Exudates*; *SDH*)	Recchiuti et al. (2011), Pan et al. (2014)	1.08	0.589
miR-146-3p	OA Inflammatory pain (DRG; SDH; synovial tissue)	Yamasaki et al. (2009), Li et al. (2011), Recchiuti et al. (2011), Li et al. (2013)	1.07	0.589
miR-483-3p	OA Cancer pain (DRG; Articular cartilage)	Iliopoulos et al. (2008), Bali et al. (2013)	1.02	1.000
miR-218a-1-3p	Neuropathic pain Cancer pain (DRG)	von Schack et al. (2011), Bali et al. (2013)	0.98	0.937
miR-106b-5p	Joint damage/inflammation (Articular cartilage)	Tao et al. (2017)	0.97	1.000
miR-124-3p	Inflammatory pain (CNS; SDH)	Nakanishi et al. (2010), Willemen et al. (2012), Kynast et al. (2013)	0.94	0.818
miR-183-5p	Neuropathic pain Inflammatory pain (*DRG*)	Bai et al. (2007), Aldrich et al. (2009)	0.94	0.485
miR-195-5p	Neuropathic pain Inflammatory pain (DRG; SDH)	Yu et al. (2011), Shi et al. (2013)	0.94	1.000
miR-664-3p	CRPS (Blood)	Orlova et al. (2011)	0.87	0.177
miR-26a-5p	Joint damage (Articular cartilage)	Iliopoulos et al. (2008)	0.86	0.310
miR-7b	Neuropathic pain Bone disorder (Osteoclasts; DRG)	Sakai et al. (2013), Dou et al. (2014)	0.83	0.240

^a^ Values represent the fold change of the OA and Naïve groups (least square means ± 95%) for miRNA expression normalized against housekeeping miR-191.

^b^ The *P*-value was determined with a Mann Whitney *U* test. Bold value highlights statistical significance.

*miRNA*, micro-RNA; *OA*, osteoarthritis; *SDH*, spinal dorsal horn; *DRG*, dorsal root ganglion; *CNS*, central nervous system; *CRPS*, complex regional pain syndrome.

### 2.3 Structural joint evaluation

#### 2.3.1 Induced-OA model in rats using the MI-RAT protocol involves macroscopic and histological lesions in the stifle structures

**FIGURE 6:**
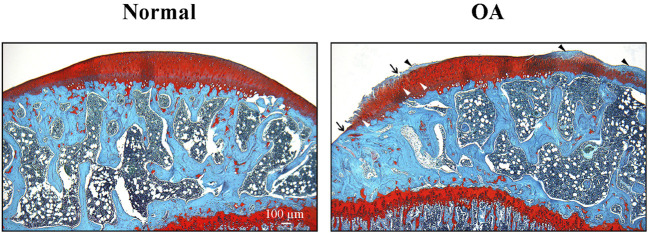
Representative cartilage of Naïve and osteoarthritis (OA) rats. Photomicrographs of representative histological sections (stained with hematoxylin and eosin, and Safranin-O/Fast green) of tibial plateaus of Naïve **(A)** and OA **(B)** rats. Arrows indicate cartilage erosion, black arrowheads the lost of proteoglycans and white arrowheads the presence of cell clusters. Original magnification ×40. Scale bar: 100 µm.

The CCLT – DMM joint instability surgery and calibrated slight exercise induced significant (*P* < 0.001) cartilage damages in the right stifle (35.77% ± 9.00% cartilage lesion score) compared (two-sided Mann-Whitney-Wilcoxon test) to the Naïve group (Table 3). Histological alterations of cartilage in OA rat stifle joints appeared to be mainly attributed to increased chondral lesions, matrix proteoglycan loss, and enhanced cluster formation (Figure 6).

**TABLE 3:** Macroscopic assessment of cartilage lesions and histological modified Mankin score (mMs) in percentage (%) of cartilage alterations of the tibial and femoral (medial and lateral) right stifle at sacrifice (D56).

		Naïve	(*n* = 6)	OA	(*n* = 12)	
Macroscopy	^b^(%)	Mean (SEM)	Median (min-max)	Mean (SEM)	Median (min-max)	^c^ *P*-value
*Total score*		3.68 (2.92)	4.40 (0.00–9.02)	35.77 (9.00)	34.91 (13.56–62.94)	< 0.001
Histology ^a^(mMs ***)***	^b^(%)	Mean (SEM)	Median (min-max)	Mean (SEM)	Median (min-max)	^c^ *P*-value
Chondral lesions		1.00 (1.77)	1.00 (0.00–2.00)	17.17 (8.16)	15.50 (10.00–29.00)	< 0.001
Proteoglycan loss		1.67 (0.96)	1.50 (1.00–3.00)	10.67 (5.40)	11.50 (5.00–16.00)	< 0.001
Cluster formation		0.17 (0.41)	0.00 (0.00–1.00)	4.42 (2.10)	4.00 (3.00–6.00)	< 0.001
Chondrocytes loss		0.00 (0.00)	0.00 (0.00–0.00)	1.58 (2.80)	2.00 (0.00–6.00)	0.020
Total score		2.83 (0.98)	2.50 (2.00–4.00)	34.25 (11.06)	33.50 (18.00–52.00)	< 0.001

^a^ Measures were obtained for the two groups in percentage of cartilage alterations for the histological modified Mankin score (mMs) of both the medial and lateral sides of the tibia and femur in the right stifle at sacrifice (D56).

^b^ Mean, standard error of the mean (SEM), median, minimum, and maximum of the total score (summation of the four compartments) are expressed in percentage of alteration.

^c^ MI-RAT model caused statistically significant apparent cartilage lesions (*P* < 0.001) perceptible at histology and macroscopy assessment 56 days after the surgery as determined by Mann-Whitney *U* testing.

## 3 Discussion

The reproducibility crisis in scientific research, particularly in the pain field, has raised doubts about the translational reliability of efficacy data from animal disease models (Klinck et al., 2017). It is crucial for animal models to closely mimic clinical conditions, including subjects used, disease induction methods, and the validity and limitations of outcome measurements (Klinck et al., 2017), yet there remains a lack of standardization design in animal model. The ideal model should be reliable, valid, and highly translational (Little and Smith, 2008), but how can the reliability and translational relevance of OA pain models be enhanced to better reflect clinical outcomes?

To address this issue, our lab focused on OA rat models over the past decade, assessing psychometric validity (repeatability and inter-rater reliability) of various functional outcomes, including biomechanical measures, operant behaviors, and sensory sensitization. Different acclimatization protocols and environmental influences (gender, observer experience, circadian cycle, exercise) were tested (Otis et al., 2016). Using the intra-articular MIA model, specificity and sensitivity of functional outcomes like PWT-assessed sensory hypersensitivity and spinal neuropeptides were evaluated, along with treatment responsiveness to pharmacological treatments (Otis et al., 2016; 2017; 2019). Recognizing limitations in the MIA model, surgical OA pain models, particularly the CCLT–DMM model, were explored for their effectiveness in inducing structural and functional alterations and spinal neuropeptidomics (Gervais et al., 2019).

Our recent studies demonstrated the relevance of using ovariectomized animals in the MI-RAT model for studying central sensitization process, showing significant analgesic effects with 17β-estradiol supplementation (Keita-Alassane et al., 2022). Adding a calibrated exercise program post-OA induction standardized structural alterations, resulting in a pain and sensory profile closer to human OA (Otis et al., 2023). The exercise MI-RAT group showed reduced mechanical pain hypersensitivity and lower levels of pro-nociceptive spinal peptides compared to the sedentary group. This effect may involve reinforced descending EIC, supported by increased spinal concentration in SST, Met-ENK, and Leu-ENK. Additionally, exercise increased BK, possibly due to joint manipulation (Meini and Maggi, 2008; Otis et al., 2023). These findings confirmed the effectiveness of incorporating slight exercise into the surgical OA model, enhancing its translatability and responsiveness to multimodal pharmacological treatments, closer to clinical OA (Otis et al., 2023).

Therefore, the aim of this study was to pursue the refinement of the MI-RAT model by validating a panel of pain assessment methods, including functional neuropeptidomics, neuroepigenetics, and innovative QST applied to the experimental MI-RAT model, in order to characterize OA pain with great validity and reliability. The results obtained provided valuable insights: neuroepigenetics appears to be activated by the development of somatosensory sensitization and its complex facilitatory/inhibitory endogenous control in the MI-RAT OA model. Therefore, the MI-RAT model induced significant structural damage, coinciding with nociceptive sensitization, as evidenced by several factors: ipsilateral weight shift towards the contralateral hind limb (asymmetry index) at D7 and D35; mechanical pain hypersensitivity observed from D7 and persisting until D56; central sensitization becoming apparent at D21; and enhanced EIC noted with a higher CPM rate at D7, D21, and D35. The somatosensory profile alterations observed in OA rats were characterized, even at D56, by an increase in pro-nociceptive neuropeptides SP and BK, alongside augmented expression of spinal miR-181b at D56.

First, functional pain outcome evaluations indicated that the MI-RAT OA model induced biomechanical (SWB) and sensory sensitization (static and dynamic QST) alterations associated with OA development. Significant ipsilateral to contralateral weight shift on SWB was observed in OA rats from the initial assessment (D7) post-OA induction, lasting until D35. However, during the subsequent 3 weeks (D49 and D56 timepoints), MI-RAT rats concealed the biomechanical pain phenotype, suggesting an attenuated perception of discomfort of the OA-induced limb, whereas in the previous timepoints, they alleviated the noxious sensation by reducing applied weight (Gervais et al., 2019). The standing on hind limbs, especially with an OA-induced stifle, appeared painful or uncomfortable for the rats. Therefore, the observed spontaneous (non-evoked) pain in SWB assessment can be interpreted as biomechanical allodynia, indicating initial sensory hypersensitivity countered by reinforced EIC, ultimately leading to condition normalization 7 weeks post-OA induction.

Secondly, the persistent decrease in the right PWT, alongside lower static QST values, throughout the whole follow-up indicated the presence of a centralized sensitization. Mechanical pain hypersensitivity in the plantar region of the ipsilateral hind limb is secondary to the damaged stifle joint, suggesting referred pain, similar to observations in OA patients (Harvey and Dickenson, 2009). Dynamic QST results revealed a significantly lower number of RMTS for OA rats at D21, representing a phenotypical form of central sensory hypersensitivity (Woolf, 2011; Guillot et al., 2014). In naturally affected OA cats, RMTS assessment has shown specificity to the OA condition and sensitivity to anti-nociceptive tramadol, but not NSAID meloxicam (Monteiro et al., 2016; Monteiro et al., 2017). Temporal summation, widely used to explore spinal cord excitability, reflects the early phase of neuronal windup, considered intrinsic to CNS changes in pathological pain. Described as activity-dependent facilitation, temporal summation evaluates conscious perception of centralized sensitization, contrasting with PWT (static QST), which assesses sensory-reflexive hypersensitivity (Guillot et al., 2014). Central sensitization mechanisms include various biochemical processes such as increased spinal release of pro-nociceptive neurotransmitters and neuromodulators, and increased excitability of postsynaptic neurons (Woolf, 1996). Neurophysiologically, temporal summation occurs when a presynaptic neuron releases neurotransmitter(s) multiple times, exceeding the postsynaptic neuron’s threshold and inducing excitability (Woolf, 1996; Woolf, 2011). To the authors knowledge, this is the first report of a dynamic QST method to an experimental OA rat model, revealing a spinal windup phenomenon likely to manifest around 3 weeks after OA induction by CCLT – DMM surgery. This is promising avenue as the authors consider bringing technological modifications to the RMTS methodology to improve the metrological properties (specificity, sensitivity, and reliability) of the method in future assessments of the MI-RAT OA model.

Finally, pain endogenous inhibitory modulation was evaluated by applying a painful CS before conducting a second static QST, based on the concept of “pain inhibiting pain” as a measure of pain perception (Yarnitsky et al., 2010; Youssef et al., 2016). This concept is based on activation of diffuse noxious inhibitory control resulting in higher pain threshold (lower sensitivity) as a functional CPM process (Le Bars et al., 1979; Yarnitsky et al., 2010). Our results indicated that CPM resulted in a 15.94% increase in post-CS PWT at baseline (unpainful) and revealed a group effect, with a more functional CPM rate observed in the OA rats throughout the entire follow-up. Moreover, at D7, D21, and D35, pain perception in OA rats was significantly modulated by a higher CPM rate, reaching a peak at D21 with a 90.37% increase in CPM intensity. At the same time (D21), there was also a significantly higher rate of positive CPM responders in OA group, aligning with the observed central sensitization noted by RMTS in the MI-RAT model. This is further explained and supported by previous QST assessments in this study, indicating that OA rats from the MI-RAT model are more painful at those timepoints, necessitating the activation of diffuse noxious inhibitory control. The CPM paradigm is currently an effective and validated somatosensory test used to identify humans suffering from chronic pain related to OA (Arant et al., 2022).

All four functional pain outcome assessments indicated nociceptive centralized sensitization and hyperexcitability processes in OA rats, predominantly observed from D7 to D35. However, at D49 and D56, only PWT, a more sensitive outcome, demonstrated these effects. During this period, hyperexcitability was progressively counteracted by efficient EIC, reducing the demand on CPM functionality, allowing OA-affected rats to conceal their biomechanical imbalance. The decline in centralized sensory sensitization, associated with reinforced EIC, was demonstrated previously by calibrated slight exercise post-OA induction in rats, compared to sedentary rats with the same surgical (CCLT – DMM) model, with a reduction, at D56, in pro-nociceptive tachykinins and an increase in anti-nociceptive SST and enkephalins (Otis et al., 2023).

Neuropeptide analysis 56 days post MI-RAT induction supports functional pain outcomes. Glutamate and aspartate act as excitatory neurotransmitters in the somatosensory system, persistently activating post-synaptic receptors and sensitizing dorsal horn neurons, leading to increased receptive field size, decreased activation threshold, and prolonged depolarization (Guillot et al., 2014; Nouri et al., 2018). Conversely, glycine and γ-aminobutyric acid (GABA) serve as chief inhibitory neurotransmitters in the somatosensory system (Ferland et al., 2011; Nouri et al., 2018). Norepinephrine’s inhibitory effects in the descending brainstem to dorsal horn pathway have a dual impact: direct activation of inhibitory GABAergic interneurons and inhibition of excitatory interneurons. Serotonin plays a key role in descending inhibitory controls, primarily from midbrain *raphe magnus nuclei*. Neuropeptides, classically considered as modulators of sensory transmission, are categorized as either excitatory or inhibitory compounds. Neurokinin A, SP and CGRP are found in intrinsic neurons of the spinal dorsal horn and are released only in specific response to noxious stimuli, sufficient to elicit sustained discharges of unmyelinated C-fibers in the superficial layers (Nouri et al., 2018). Vesicles containing mature neuropeptides undergo exocytosis at the synaptic cleft, in response to noxious stimuli, facilitatory/inhibitory controls, influencing neurotransmission and modulating neuronal signals. These peptides, rather than acting as synaptic transmitters, diffuse in the dorsal horn, potentially influencing multiple synapses distant from their release point, contributing to final sensory sensitization signal (Nouri et al., 2018). Spinal inhibitory neuropeptides include SST, enkephalins, and possibly dynorphin, found in intrinsic neurons of the dorsal horn (local circuit) and fibers descending from various brainstem *nuclei*. Sensory sensitization may result from long-term potentiation, reduced GABAergic or glycinergic inhibitory neurotransmission (disinhibition), intrinsic plasticity in dorsal horn neurons, and changes in low threshold mechanoreceptive Aβ afferents. Many excitatory interneurons contain SST, and the resulting increase in the release of this peptide may hyperpolarize nearby inhibitory interneurons, exerting a disinhibitory effect (Prescott, 2015). At D56, CGRP concentration was lower than expected, indicating possible establishment and fluctuation of nociceptive sensitization, partly influenced by SP. In a dose-response comparative study of the chemical MIA model in rats (Otis et al., 2017), SP, CGRP, BK, SST, and transthyretin exhibited a correlation with cartilage lesions and functional assessments. These markers were also highly sensitive in our original neuropeptidomics screening across various pain animal models (Rialland et al., 2014a; Rialland et al., 2014b). Indeed, SST uniquely demonstrated a dose-effect with MIA intra-articular injection (Otis et al., 2017) and paralleled structural alterations induced by different surgical OA models (Gervais et al., 2019), highlighting its heightened sensitivity in pain detection. Moreover, SP, SST, and transthyretin mimicked the beneficial effects observed on functional assessments for different analgesics, namely intra-articular lidocaine (Otis et al., 2016), systemic pregabalin, morphine and carprofen (Otis et al., 2019), suggesting a higher sensitivity of these biomarkers in treatment responsiveness.

Somatostatin exhibits anti-inflammatory and anti-(or pro-) nociceptive effects through interactions with other neuropeptides, including SP and CGRP (Pinter et al., 2006). The increased SST release in inflammatory conditions, along with other neuropeptides, indicates the potential for hyperexcitability induction (Gervais et al., 2019). Although SST levels were elevated in the OA group, the difference with the Naïve group was not statistically significant (*P* = 0.065). Another noteworthy neuropeptide in OA pain is BK, a kinin family peptide released during tissue injury and inflammation, acting on B_2_ and injury-induced B_1_ G-protein-coupled receptors expressed on peripheral terminals of primary neurons (Ferreira et al., 2002; Wang et al., 2005). Interaction of BK with other pro-inflammatory and hyperalgesic mediators, such as ion channels, prostaglandins, CGRP and other neuropeptides, highlights its pro-inflammatory and nociceptive properties (Ferreira et al., 2002; Aldossary et al., 2024). Despite being primarily considered a peripheral inflammatory mediator, BK is also involved in central pain transmission, with identified B_1_ and B_2_ receptors in the dorsal root ganglion and spinal dorsal horn modulating glutamatergic transmission (Ferreira et al., 2002; Wang et al., 2005; Kohno et al., 2008). These findings suggest that, by the end of the study, nociceptive processes were gradually coming under control, explaining the limited differences in functional pain outcomes observed at D49 and D56 (statistically significant only for PWT). Additionally, neuroepigenetic changes likely play a regulatory role in nociceptive processes, as supported by miRNA expression levels correlating with neuropeptidomic data analysis.

Circulating miRNAs hold increasing importance in clinical medicine for diagnostic, prognostic, and therapeutic stratification (Tramullas et al., 2018). Their stability, specificity, and ease of detection make them promising tools for personalized medicine, aiding in the management of chronic pain and guiding therapeutic decisions. Our objective was to identify potential differences in spinal miRNA expression between a Naïve group and an OA group (MI-RAT model) in rat spinal cord tissue. Our expectations, supported by the validated OA model (Gervais et al., 2019; Keita-Alassane et al., 2022; Otis et al., 2023), were partially met, revealing limited differences 56 days post-OA induction. With functional alterations more persisting up to D35, would they be linked to higher neuropeptidomics and neuroepigenetics changes at this time? However, by D56, observed neuropeptidomic changes were potentially declining. In the few past years, studies have explored miRNA expression in chronic, neuropathic, and inflammatory pain conditions. Most studies utilized chemical models like intra-articular MIA injection (Li et al., 2011), inflammatory models (complete Freund’s adjuvant (Bai et al., 2007; Pan et al., 2014), formalin (Kynast et al., 2013), interleukin-1β (Willemen et al., 2012), bone-cancer pain (Bali et al., 2013), or neuropathic models (Aldrich et al., 2009; Nakanishi et al., 2010; Kusuda et al., 2011; von Schack et al., 2011; Yu et al., 2011; Bhalala et al., 2012; Sakai et al., 2013; Sakai and Suzuki, 2013; Shi et al., 2013), to reveal neural miRNA expression alterations. In a neuropathic pain study (Tramullas et al., 2018), miR-30c-5p showed upregulation in the spinal dorsal horn 2 weeks post sciatic nerve injury. Intriguingly, this miRNA was the only one significantly dysregulated following qPCR validation, with a modest fold change from next-generation sequencing. Specifically for OA pain in rats, a surgical model (medial meniscus transection) was tested for behavioral (secondary tactile allodynia via static QST), structural, and neuroepigenetic alterations over 8 weeks (Li et al., 2013). This study identified downregulation of miR-146a and miR-183 clusters in the dorsal root ganglia and spinal cord at weeks 4 and 8 (but not 2) post-OA induction, while sensory sensitivity persisted throughout the follow-up (Li et al., 2013). Moreover, OA establishment in human patients or mice models has correlated with miRNA dysregulation in tissues other than the dorsal root ganglia or spinal cord, such as joint tissues (cartilage, bone, synovium) (Iliopoulos et al., 2008; Yamasaki et al., 2009; Song et al., 2013; Nakamura et al., 2016; Tao et al., 2017; Nakamura et al., 2019). Using a DMM model in mice, it was not possible to demonstrate serum miRNA dysregulation between the OA group and the control group, even though DMM mice showed significant histological signs of cartilage degradation (Kung et al., 2017).

In the current study, only one potential neuroepigenetic biomarker demonstrated a different expression between the OA and Naïve groups. Circulating miR181b-5p, identified as elevated in patients with myalgic encephalomyelitis/chronic fatigue syndrome (Nepotchatykh et al., 2020), showed increased expression in the spinal cord of OA rats. Inversely, repressed miR-181b-5p has been reported to participate in the pathogenesis of inflammation and neurological diseases (Lu et al., 2019). The present finding is noteworthy for various reasons in OA research. Structurally, miR-181b and its closely related family member, miR-181a, have been implicated as potential mediators of cartilage degeneration in OA facet and knee joints (Song et al., 2013; Nakamura et al., 2016; Nakamura et al., 2019). Members of the miR-181 family are associated with the upregulation of catabolic matrix metalloproteinase-13, release of inflammatory mediators, and cartilage degradation (Nakamura et al., 2016). Functionally, the miR-181 family is of interest in OA, as it has been linked to GABAergic regulation (Zhao et al., 2012; Sengupta et al., 2013). The overexpression of miR-181a and miR-181b in the spinal cord of a visceral inflammatory model has been demonstrated, associating the upregulation of miR-181b with a downregulation of GABA_Aα-1_ receptor subunit mRNA and protein (Sengupta et al., 2013). The majority of neurons (>95%) in the spinal dorsal horn are local circuit interneurons releasing neuromodulatory substances such as enkephalin, glycine, and GABA (Prescott, 2015; Nouri et al., 2018), and it could be hypothesized that miR181b participates in the spinal dorsal horn inhibitory/facilitatory balance in nociceptive neuromodulation, as suggested by our neuropeptidomics results, specifically concerning SST and transthyretin, both involved in inhibiting neuronal activity. Few years ago, transthyretin knockdown has been associated with decreased GABA_A_ receptor expression (Zhou et al., 2019), where higher level of functional pain and mechanical pain sensitivity were observed in different pain models associated with spinally decreased, and increased, transthyretin, and SST, respectively (Rialland et al., 2014a; Rialland et al., 2014b; Otis et al., 2016; Otis et al., 2017; Gervais et al., 2019; Otis et al., 2019; Otis et al., 2023). Moreover, SST interneurons in the brain were shown to inhibit excitatory transmission through GABA_B_ receptor astrocytic and presynaptic activation (Shen et al., 2022). Thus, these small molecules hold promise as pain assessment biomarkers, reflecting dysfunction in pain processing at different levels (transmission, modulation, perception, etc.), therefore enhancing our comprehension of various chronic pain states and facilitating the development of novel analgesics. The present discovery of miR-181b-5p in OA pain modulation (inhibition of GABAergic transmission) and the recognized involvement of the miR-181 family in OA structural alterations will require further clarification in the future. Research into the specific mechanisms by which miR-101b-5p influences pain pathways could provide valuable insights into the pathophysiology of chronic pain, and lead to the development of novel therapeutic strategies.

Macroscopic and histological assessments demonstrated moderate lesions in the right stifle of rats in the OA group. From a histological standpoint, chondral lesions, loss of proteoglycans, and cluster formation were the most significant injuries observed in the damaged joint. Hence, MI-RAT model achieved to change the hyaline cartilage to the point where 35.77% of the structure showed irregularities and erosion. Increased cluster formation leads to a dysregulation of cartilage homeostasis, that affects other joint structures, and is more pronounced in mature alterations (Lotz et al., 2010). These alterations, along with the significant loss of proteoglycans–crucial hydrophilic substances enabling the absorption of mechanical impacts–and chondrocytes, typically initiate substantial changes in all articular components, triggering processes associated with OA pain (Xia et al., 2014). Notably, there is a remarkable consistency in stifle histological alterations, static QST, and targeted neuropeptides spinal concentration between the current study and our previous one (Otis et al., 2023), employing the same MI-RAT OA model. This underscores its excellent reproducibility and validity. The differences in miRNA expression found in OA subjects do not necessarily reflect the extent of cartilage degeneration, but perhaps some other aspects of joint pathology (Kung et al., 2017). Thus, the structure/function (including pain) correlation in OA would require further investigations, particularly for epigenetics considering that miR-181a appears to be involved in OA cartilage degradation (Song et al., 2013; Nakamura et al., 2016; Nakamura et al., 2019). A serial assessment of joint structural alteration developed in this MI-RAT model in relation to pain functional behaviors and measures of spinal targeted pain neuropeptides as well as joint and spinal epigenetics would precise the structure/function inter-relationship. Too often, experimental rodent models use focused on molecular and pathophysiological joint structural changes as indicators of successful intervention, but without investigating pain behavior. The refinement of the MI-RAT OA model, including standardized procedures (peri-operative analgesia, anesthesia, surgical procedure, enrichment, calibrated exercise, behavioral outcome measures) with the emergence of QST applications in animals, as well as neuropeptidomic and epigenetic biomarkers bonify the validity of MI-RAT for studying both structural (joint) and neurophysiological changes associated with the OA model.

This study demonstrated that OA pain development in the MI-RAT model is strongly linked to centralized sensitization and enhanced EIC activation, evidenced by concurrent changes in pain phenotype, neuropeptidomic and neuroepigenetics biomarkers. Of utmost importance, homogenous joint structural lesions were observed, permitting us to compare other outcomes measures. Functional pain assessments and neuropeptidomic analysis at D56 indicated the development of centralized sensitization, with the CNS gradually gaining control over (hyper)nociceptive inputs despite heightened inflammation signals. The complete functional pain platform included non-evoked mechanical sensitivity (through SWB) waning at D35, as well as evoked static QST (through PWT) persisting up to D56, whereas dynamic QST highlighted the involvement of endogenous facilitatory (through RMTS) and inhibitory (through CPM) controls during the initial timepoints, reaching a peak at D21 and fading at D35. Neuroepigenetic analysis showed elevated spinal expression of miR-181b-5p following inflammatory and nociceptive inputs from stifle joint lesions. Overexpression of spinal miR-181b can repress the GABAergic central inhibitory system. This repression disrupts the balance between excitatory and inhibitory neurotransmission in the spinal cord, potentially contributing to hyperexcitability and enhanced nociceptive signaling, thus exacerbating pain perception and chronic pain conditions. This preliminary study had limitations, including potential interference from the exercise protocol, a limited sample size effect, and the need for additional molecular analysis validation at various time points and tissues in future studies. Nonetheless, the present study highlights the potential use of neuroepigenetic analysis, combined with pain phenotype changes and functional targeted neuropeptide outcomes, to enhance our understanding of constituents in OA pain mechanisms and advance translational research.

## 4 Materials and methods

### 4.1 Ethics statement

Institutional Animal Care and Use Committee of Université de Montréal approved the protocol (#Rech-1766) which was conducted in accordance with principles outlined in the current Guide to the Care and Use of Experimental Animals published by the Canadian Council on Animal Care and the Guide for the Care and Use of Laboratory Animals published by the US National Institutes of Health.

### 4.2 Animals

The study was conducted on adult ovariectomized female (*n* = 24) Sprague-Dawley rats (Charles-River Laboratories, Saint-Constant, QC, Canada), as previously validated (Keita-Alassane et al., 2022), weighing between 230 and 250 g at the beginning of the study. Rats were housed under regular laboratory conditions and maintained under a 12-h light-dark cycle with food and water provided *ad libitum*. Animals were randomly divided in two groups: Naïve (*n* = 12) and surgically induced OA (*n* = 12). Subjects belonging to the same group were paired and caged together. Body weight (g) was obtained weekly.

### 4.3 Montreal Induction of Rat Arthritis Testing (MI-RAT) model

#### 4.3.1 Anesthesia and analgesia

On the day of the surgical intervention (D0), rats were placed in an induction box and anesthetized with an isoflurane-O_2_ mixture (IsoFlo^®^, Abbott Animal Health, Montreal, Québec, Canada). Anesthesia was maintained with a 2% isoflurane-O_2_ mixture *via* a face mask and a non-rebreathing Bain system. After anesthesia induction, a single intramuscular premedication injection of 1.0 mg/kg of Buprenorphine SR™ (Chiron Compounding Pharmacy Inc., Guelph, ON, Canada) was administered to provide approximately 72 h of analgesic coverage (Gervais et al., 2019; Keita-Alassane et al., 2022; Otis et al., 2023). A periarticular block of 0.25% bupivacaine (Marcaine^®^, McKesson Canada, St.-Laurent, Québec, Canada) at a dose of 0.05–0.1 mL per stifle (<1 mg/kg) was given at the end of the surgical procedure (Gervais et al., 2019; Keita-Alassane et al., 2022; Otis et al., 2023).

#### 4.3.2 Surgical OA-induction

Animals were placed in dorsal recumbency, and their right hind limb was prepared using aseptic techniques. The surgical CCLT – DMM procedure was performed as previously described (Gervais et al., 2019) and validated (Keita-Alassane et al., 2022; Otis et al., 2023): skin incision, medial parapatellar arthrotomy, lateral patella luxation, DMM (by transection of the cranio-medial meniscotibial ligament) and CCLT, patellar reduction, and surgical site closure in sequential planes. All animals successfully completed the study, and there was no complication following the surgical procedure.

#### 4.3.3 Exercise protocol

The MI-RAT model included a regular exercise protocol, a 10-min running period on a motor-driven treadmill (IITC Life Science Inc., Woodland Hills, CA, United States) for rodents, at a constant speed of 18.3 cm/s, on three non-consecutive days a week for 8 weeks (Otis et al., 2023). This protocol was associated with surgical joint instability, aiming to minimize variability in functional OA pain outcomes and structural joint OA alterations, as observed in the MIA rat model (Otis et al., 2016).

### 4.4 Functional pain assessment

It involved biomechanical distribution through static weight-bearing (SWB), a non-evoked behavioral measure of musculoskeletal pain, and a somatosensory profile using a QST protocol recognized as a behavioral expression of nociceptive sensitization (Cruz-Almeida and Fillingim, 2013). Rats were acclimatized to the evaluation environment at D–14, D–7, D–5, and D–3, as per previous rat validation studies (Otis et al., 2016; Keita-Alassane et al., 2022; Otis et al., 2023). One day before OA induction, baseline values for functional assessments were established. Assessments were repeated at D7, D14, D21, D35, D49, D56. Observers (female) remained completely blinded to OA induction and the experimental design and operated during daylight (Otis et al., 2016).

#### 4.4.1 Static weight-bearing (SWB)

An Incapacitance Meter^®^ (IITC Life Science Inc., Woodland Hills, CA, United States) was employed to assess SWB distribution between the right and left hind limbs (Otis et al., 2016; Otis et al., 2017; Otis et al., 2023). The weight (force) applied by the animal for each hind limb was measured in grams but expressed as a percentage of total body weight (%BW) to normalize the data for each animal before calculating the asymmetry index value. Measurements were obtained over a 3-s period simultaneously for each limb, and triplicate readings were taken at each timepoint. Contralateral report (SWB asymmetry index) was assessed by calculating the weight report from the ipsilateral to the contralateral hind limb, as follows:

$$\text{SWB asymmetry index }\left(\%\right)=\left(\left(\%{\text{BW}}_{\text{right}}\;–\;\%{\text{BW}}_{\text{left}}\right)\;/\;\left[\left(\%{\text{BW}}_{\text{right}}+\%{\text{BW}}_{\text{left}}\right)\;*\;0.5\right]\right)\;*\;100$$

#### 4.4.2 Quantitative sensory testing (QST)

Static QST assesses the sensory threshold or the rating of a single stimulus (Arendt-Nielsen and Yarnitsky, 2009). In this study, it involved secondary mechanical pain sensitivity using an electronic von Frey Esthesiometer^®^ with a propylene probe Rigid Tip^®^ of 0.7 mm^2^ surface, 28G (IITC Life Sciences Inc., Woodland Hills, CA, United States) to determine the paw withdrawal threshold (PWT) of each hind paw. Static QST was performed as described previously (Otis et al., 2016; Otis et al., 2017; Otis et al., 2023). The peak force was recorded in grams, and a cut-off value was set at 100 g. Both hind paws were evaluated alternately, and triplicate measures were taken for each, with 60-s intervals between stimuli for each animal.

Dynamic QST assesses the response to a number of stimuli (Arendt-Nielsen and Yarnitsky, 2009; Guillot et al., 2014; Mackey et al., 2017), providing the opportunity to investigate the central processing of incoming nociceptive signals (Guillot et al., 2014; Mackey et al., 2017). In this experiment, dynamic QST was evaluated by measuring the response to mechanical temporal summation (RMTS) and conditioned pain modulation (CPM).

The RMTS was assessed by inducing repeated sub-threshold intensity mechanical stimuli (TopCat Metrology Ltd., Cambs, United Kingdom) previously validated in OA cats (Guillot et al., 2014). The mechanical stimulation, set at a predetermined and steady 2N intensity (0.4 Hz), was applied through a hemispherical-ended metallic pin (2.5 mm diameter, 10 mm length) mounted on a rolling diaphragm actuator, adapted from a validated mechanical threshold testing system (Dixon et al., 2010). The mechanical stimulator was positioned on the rat’s back and secured by a narrow strap passing under the thorax just behind the front limbs. Animals had freedom of movement in the cage before each session and during testing. Normal behavior of the rat wearing the device in the cage was observed for 5 min. Sessions were stopped by the evaluator as soon as clear disagreeable reaction was observed (e.g., vocalization, agitation, biting at the band) or when the cut-off number (*n* = 30) was reached and noted as the number of stimuli. Each assessment included a description of the rat’s behavior. Due to its preliminary validation, RMTS was measured at a limited number of timepoints (Baseline, D21, D35, and D56).

The CPM paradigm serves as a psychophysical experimental measure of the pain endogenous inhibitory pathway (Yarnitsky et al., 2010), associated with diffuse noxious inhibitory control (DNIC) in humans (Youssef et al., 2016), originally demonstrated in rats (Le Bars et al., 1979). It involves the application of a conditioning stimulus (CS) to decrease pain perception following an initial noxious stimulus (Mackey et al., 2017). Dysfunction of the descending EIC was shown in dogs with primary bone cancer using CPM (Monteiro et al., 2018). In rats, the functional CPM PWT rate was measured with a dynamic CS induced by clipping the left ear with a curved Bulldog serrifine clamp (50 mm in length, duration of 1 min) before performing a second static QST (post-CS). The difference (delta) of post-CS minus pre-CS was used to calculate the CPM rate. Functionality of the CPM response was determined as looking at positive responders to CS. Individual CPM rate was calculated as follows:

$$\text{CPM rate }\left(\%\right)=100+\left[\left(\left(\text{PWT}\;\text{post-CS}\;-\text{PWT}\;\text{preCS}\right)\;/\;\left(\text{PWT}\;\text{pre-CS}\right)\right)\;*\;100\right]$$

A rat was considered as a positive responder if its CPM rate in ipsilateral hind PWT was higher than 100% (considered as no change) during the follow-up (post-OA induction) at each timepoint.

### 4.5 Molecular analysis

#### 4.5.1 Euthanasia and spinal cord collection

After the final functional evaluation day (D56), euthanasia was carried out by transection of the cervical spine using a guillotine following a 4%–5% isoflurane overdose. Immediately after decapitation, the entire spinal cord was collected using a saline flush technique (Ferland et al., 2011; Otis et al., 2016; Otis et al., 2017; Gervais et al., 2019; Otis et al., 2019; Keita-Alassane et al., 2022; Otis et al., 2023). Samples were quickly snap-frozen in cold hexane, stored individually, and kept at −80°C for subsequent neuroepigenetic (half samples) and neuropeptidomic (half samples) analyses.

#### 4.5.2 Neuropeptidomic analysis

All chemicals were obtained from Sigma-Aldrich (Oakville, ON, Canada), unless specifically indicated. Tissue processing is a crucial step in preserving neuropeptides from *in situ* degradation (Beaudry, 2010). Rat spinal cords (*n* = 6 in each group) were individually weighed precisely, homogenized, and processed as previously described (Otis et al., 2019; Keita-Alassane et al., 2022; Otis et al., 2023). Peptides were then extracted using a standard C18 solid-phase extraction protocol as also published formerly (Otis et al., 2019).

Quantification of extracted neuropeptides from spinal cord homogenate was achieved by mass spectrometry coupled with a liquid chromatography system. Chromatography was performed using a gradient mobile phase along with a microbore column Thermo Biobasic C18 100 × 1 mm, with a particle size of 5 μm (Vanquish FLEX UHPLC^®^ system, Thermo Scientific, San Jose, CA, United States) as described previously (Otis et al., 2019; Otis et al., 2023). Mass spectrometry detection was performed using a Q-Exactive Orbitrap Mass Spectrometer^®^ (Thermo Scientific, San Jose, CA, United States) interfaced with an UltiMate 3000^®^ Rapid Separation UHPLC system using a pneumatic-assisted heated electrospray ion source. Peptide quantification for SP, CGRP, BK, and SST was determined using stable isotope labelled internal standard peptides and expressed in fmol/mg of spinal cord homogenate, as previously described (Ferland et al., 2011; Otis et al., 2016; Otis et al., 2017; Otis et al., 2019; Keita-Alassane et al., 2022; Otis et al., 2023).

#### 4.5.3 miRNA analysis: RNA extraction, miRNA screening and real-time quantitative polymerase chain reaction (RT-qPCR) assays

Total RNA was extracted from (30 mg lumbar portion) rat spinal cord samples (L5-S1) using miRCURY™ RNA isolation kit for tissues (#300115, Exiqon Inc., Woburn, MA, United States). Manufacturer protocol was followed except for the elution step that was collected in 35 μL. Quantification and quality control for total RNA samples was acquired using the total RNA Nanochip assay on an Agilent 210^®^ Bioanalyzer (Agilent Technologies Inc., Santa Clara, CA, United States).

MiRNA selection was based on a literature review englobing original studies about (miRNA OR microRNA) AND (chronic pain OR osteoarthritis OR osteoarthrosis OR degenerative joint disease) AND (chronic pain OR nociceptive OR inflammatory OR neuropathic OR cancer OR cancerous) AND (spinal cord OR central nervous system). Fourteen miRNAs were chosen, based on their yet existing or potential role in pain, for expression screening in all spinal cord samples (*n* = 6 in each group). Conditions and tissues in which selected miRNA has been described are briefly presented in Table 2 with respective references.

TaqMan^®^ MicroRNA Reverse Transcription Kit (#4366596, Applied Biosystems, Carlsbad, CA, United States) was used for reverse transcription of total RNA samples and TaqMan^®^ MicroRNA Assays kit (#4427975) was used with TaqMan^®^ Fast Advanced Master Mix (#4444556) for RT-qPCR amplification following manufacturer’s instructions. Expression levels were normalized using miR-191 as reference, as suggested by TaqMan^®^ technical guide, since its expression has been reported as consistent across several tissues (Peltier and Latham, 2008; Schwarzenbach et al., 2015), and found as not different in both groups. Fold change of miRNA expression was calculated on obtained comparative cycle threshold (Ct) RT-qPCR ratios which were calculated with an efficiency correction using the Pfaffl Method (Pfaffl, 2001).

### 4.6 Structural joint evaluation

Right stifle joints from OA (*n* = 12) and Naïve (*n* = 6) rats were collected and dissected free of muscle immediately following sacrifice at D56. The stifle joints were fixed in 10% formaldehyde solution (pH 7.4) for at least 3 days.

#### 4.6.1 Evaluation of macroscopic lesions

Examination of the right stifle for morphological changes was performed by two independent observers under blinded conditions as previously described (Fernandes et al., 1995). Macroscopic lesions of medial and lateral aspects of femoral condyles and tibial plateaus were characterized based on the surface area (size) of articular surface changes which were measured (ImageJ, U. S. National Institutes of Health, Bethesda, Maryland, United States), and expressed in mm^2^. For each compartment, the macroscopic cartilage lesions size was reported on the total compartment size and expressed in percentage of cartilage alteration. The total score was emitted as the mean (standard error of the mean), median (min-max) of the four compartments for both groups.

#### 4.6.2 Histological analysis

Joint tissues were decalcified and embedded in paraffin for histological evaluation. Serial sections were cut with a thickness of 5 μm for each stifle after a hematoxylin and eosin, or Safranin-O/Fast Green staining. Medial and lateral femoral condyles, as well as medial and lateral tibial plateaus were analyzed. Articular lesions were graded on a scale using a table modified from Mankin’s score (mMs) (Colombo et al., 1983; Gerwin et al., 2010; Otis et al., 2023) by an independent observer blinded to the study. Severity of lesions ranged from 0 to 25 for each of the four compartments of the stifle. Structural changes were scored from 0 (normal) to 10 (highest surface irregularities) to assess chondral lesions; Safranin-O staining was evaluated to identify proteoglycan loss with a scale from 0 (no loss of staining) to 6 (loss of staining in all the articular cartilage by more than 50%); clusters formation was evaluated from a range of 0 (no cluster formation) to 3 (more than 8 clusters); and loss of chondrocytes was scored on 0 (normal) to 6 (diffuse loss of chondrocytes) scale. The sum of all four compartment scores was calculated (maximum 100) and was expressed in percentage of cartilage alterations (Otis et al., 2023).

### 4.7 Statistical analysis

All statistical analyses were conducted using IBM^®^ SPSS^®^ Statistics Server version 26.0 (New York City, NY, United States), with a threshold alpha set at 5% for inferential analysis. First, the data averages of the three trials for SWB and PWT were calculated for each subject. Then, data from functional pain outcomes (SWB, static and dynamic QST) were analyzed using general linear mixed models for repeated measures (Otis et al., 2016; Otis et al., 2017; Otis et al., 2019; Keita-Alassane et al., 2022; Otis et al., 2023), and the normality of the outcomes residuals was verified using the Shapiro-Wilk test. The groups, days and their interactions (day *x* group) were considered as fixed effects, with baseline measurement as covariate and using type-3 regressive covariance structure. Data are presented as the estimate mean (least square mean (LSM)) with 95% confidence limits (inferior and superior). The number of CPM positive responders was analysed by using a Fisher’s exact test. Neuropeptides, epigenetic, macroscopic and histological joint data were analyzed using the nonparametric two-sided Mann-Whitney-Wilcoxon test.

## Data Availability

The datasets presented in this study can be found in an online repository: https://data.mendeley.com/datasets/j9ky4cpdrn/12.
